# Comparison of the effectiveness of flipped classroom and traditional teaching method on the components of self-determination and class perception among University students

**DOI:** 10.30476/JAMP.2021.89793.1385

**Published:** 2021-10

**Authors:** MAHNAZ KHAYAT, FARIBA HAFEZI, PARVIZ ASGARI, MARZIEH TALEBZADEH SHOUSHTARI

**Affiliations:** 1 Department of Psychology, Ahvaz Branch, Islamic Azad University, Ahvaz, Iran

**Keywords:** Teaching, Self-determination, Students

## Abstract

**Introduction:**

The flipped classroom instruction can be an opportunity to make educational changes in class contents, which means this model can be a way to rethink learning and the educational process. The present study aimed to investigate the effectiveness of flipped classroom and traditional teaching methods on the components of self-determination and the class perception among university students.

**Methods:**

This is an experimental study with a pre-test and post-test design and a control group. The study population comprised of all female students of Farhangian University in Ahvaz city in the academic year 2019. The sample consisted of 36 students selected by convenience sampling. We randomly divided the participants into experimental (n=18) and control (n=18) groups. The research instruments included the Basic Psychological Need Satisfaction and the Class Perception Questionnaire. The experimental group received the flipped teaching program during six 120-minute sessions once a week; however, the control group received the traditional teaching method. Data were analyzed by descriptive and inferential statistics, such as mean, standard deviation, analysis of covariance (ANCOVA), and multivariate analysis of covariance (MANCOVA).

**Results:**

The results indicated that there was a significant difference between the flipped classroom and traditional teaching (p< 0.05) on the components of self-determination and the class perception among university students.

**Conclusion:**

According to the results, the flipped teaching method had greater impacts on the components of self-determination and class perception in university students, compared to the traditional method.

## Introduction

Over the last decades, significant innovations and advancements of information technology have emerged, and technology has become a valuable part of the education process. The advent of low-price data storage networks, the effectiveness of advanced computers, and new devices such as smartphones have created new digital experiences for students, leading the new generation to change their daily lives and learning habits. Students in the new millennium are more reliant on information technology and less tolerant of common educational patterns. In other words, students have other needs and expectations of educational systems. Traditional education methods no longer meet the students' needs. In that regard, the instructors should provide more opportunities for students to participate. This thought requires a change in the traditional teachers' approach to active-learning of students, which actively involves the students in learning ( 1 ).

The flipped classroom is an alternative educational approach that emphasizes the student-centered teaching method, keeping the traditional classroom environment as a reserve. It also widely captured interests and is accepted in high education levels. Flipped classroom, as a student-centered learning method, includes several theories and methods of constructivism and active learning with education peers' help ( 2 ). The flipped classroom is a pedagogical approach in which direct instruction moves from the group learning space to the individual learning space. The resulting group space is transformed into a dynamic, interactive learning environment where the educator guides the students as they apply concepts and engage creatively in the subject matter ( 3 ). Besides, the flipped classroom is a learning environment that provides students with a variety of tools to prepare the basic knowledge as part of their homework and for classroom meetings. Teachers then employ the class time more effectively to present activities and encourage the students to practice lessons using the training content, which is an essential issue before holding classes ( 4 ).

The flipped classroom instruction can be an opportunity to make educational changes in class contents, which means that this model can be a way to rethink learning and the educational process. The teacher can also act as a learning designer. Ultimately, flipped classroom activities are determined by ideas such as learner-centered learning and theories like self-determination ( 5 ). Kheirabadi ( 6 ) indicated that the students' satisfaction and incentive enhancement, and optimization of the teaching process in terms of time management and repetitive and tedious processes prevention have been the effects of using the flipped classroom. Joshaghan Nejhad and Bagheri ( 7 ) have concluded if the flipped classroom implementation is facilitated, it is more effective than traditional teaching. Kavianii et al. ( 3 ) reported that the students were engaged with the curriculum through participation and interaction in the flipped classroom. In this method, the instructor acts as a tutor and facilitator to increase the learners' responsibility for their learning process, including what they learn and how they learn it.

Self-determination theory presents three facets: competency, autonomy, and relatedness, all of which being students' requirements. Competency relates to the students' needs to feel capable and mastery in the learning process. Autonomy is associated with the need to participate in the tasks related to the person independently. Relatedness is associated with the need to engage in tasks that allow the students to interact and communicate with their peers ( 8 ). According to Narendran et al. ( 9 ), these three psychological needs (competency, autonomy, relatedness) lead to the learners' engagement. However, when teachers, who are a part of the student's external environment, have major control over the learning process, students' sense of autonomy and competence diminishes. Instead, in active learning environments, when students properly do what they have learned independently, teachers encourage and support them, facilitating the learners' independence. Furthermore, the feeling of competence, which is the feeling of being efficient and having control over behavior, is created following the positive feedback towards improving the way of thinking and skill level of learners during team assessment in a significant and particular manner. The communication also demonstrates a sense of belonging to social society. Students are likely to experience higher communication levels in small groups' participatory classroom learning activities; therefore, they have more chances to satisfy this need ( 5 ).

Students can do their assignments individually or in groups in classrooms, solving their issues with their teacher's assistance during active learning and collaborating with others. Accordingly, along with the fact that the content provided in classrooms does not seem new to the students, reviewing the learned material will lay the foundation for more sustainable learning. This instruction method gives the students chances to review the educational content they are provided repeatedly, observing the educational videos. The flipped education proponents believe that the students have a deeper involvement with the educational materials provided in the classrooms in this teaching method, giving a better response to this type of education ( 10 , 11 ). The flipped instruction model's advantages comprise more teacher-student interaction, simultaneous feedback in the classrooms, students' self-learning concerning the need, and a better understanding of the materials ( 12 ). The flipped classroom method stems from the social theories of instruction and active learning. Consequently, participatory learning and educating peers during the class sessions lead to a more positive understanding of the contents of a course ( 13 ).

Several studies have demonstrated the positive effects of the flipped classroom approach on self-determination. As an illustration, Muir ( 14 ), Zainuddin and Perera ( 15 ), and Abeysekera and Dawson ( 16 ) indicated that the flipped classroom approach affected the students' self-determination, enhancing their incentive, as well as contribution level. Vahidi and Poushaneh ( 17 ) demonstrated that employing the flipped classroom instruction method has a significant effect on the students' metacognitive skills and educational incentive. Piri et al. ( 18 ) reported that with pre-test assistance, the flipped education method has been able to have a substantial influence on the self-directed learning variable. Accordingly, the average scores of self-directed learning students instructed via the flipped classroom method were higher. Bahmani et al. ( 19 ) figured out that the flipped classroom students took part in the activities pertinent to English practices and assignments.

Given the significance of variables such as self-determination and class perception, the present study aimed to compare the effectiveness of flipped teaching and traditional teaching on the components of self-determination and class perception among university students.

## Methods

This is an experimental study with a pre-test and post-test design and a control group. The statistical population included all female students of Farhangian University in Ahvaz city in the academic year 2019. Using the purposive sampling method, 36 students were selected and randomly divided into experimental and control groups (n=18 per group). Pre-test and post-test were performed in both experimental and control groups before and after the intervention, respectively. Inclusion criteria were being a freshman, taking statistics course, being familiar with virtual networks and the Internet, and being willing to participate in the study. Exclusion criteria were being absent for more than one session during the semester and incomplete questionnaires. In the present study, the researcher adopted the traditional and flipped statistics classroom approaches.

### Procedure

The flipped classroom was organized as follows:

1- Pre-classroom phase: In this phase, group teaching in the classroom was converted into individual teaching at home. The students watched the class electronic content from the statistics book for each session, as specified by the instructor, and answered the four-choice questions selected from the same content. To answer the questions, students were given the opportunity to watch and read electronic content whenever they wished and learned the content taught tailored to their own learning pace.
2. Attendance in classroom: In the classroom, the instructor offered a brief review of the electronic content and provided the students with additional explanations. Then, the instructor discussed the concerned four-choice questions and removed the ambiguities. In the classroom, the instructor also formed groups of three and four (the students could select their groupmates) and presented the questions extracted from the statistics book to the groups to be discussed in groups. When the students were doing the exercises, the instructor was walking around the classroom to answer their possible questions with the help of their groupmates.

The traditional classroom was organized as follows:

In the traditional classroom, the lesson was lectured, and the course instructor presented the materials and exercises in PowerPoint slides. The students in the classroom often listened and took notes. In the classroom, there were some exercises and discussions about the book content, for which there were often not enough time to do them; hence, the students were supposed to do them at home.

### Research instruments

**Basic Psychological Need Satisfaction:** This questionnaire was developed by La Guardia et al. ( 20 ) and consisted of 21 items, scored on a seven-point Likert scale ranging from "Completely false" to "Completely true". This scale measures three subscales of autonomy (7 items), competency (6 items), and relatedness (8 items). The scores of each subscale range from 7 to 49, and the total score is obtained by adding the scores of all the items. A higher score in each scale indicates a higher level of satisfaction. Arfaa Baluchi et al. ( 21 ) reported an alpha Cronbach coefficient of 0.88 for the questionnaire. Deci et al. ( 22 ) reported a Cronbach’s Alpha coefficient of 0.83 for the reliability of this questionnaire. In the present study, Cronbach's alpha coefficient was 0.80 for the questionnaire.

**Class Perception Questionnaire:** This questionnaire was made by Fraser et al. ( 23 ) and has seven subscales and 54 items. The items separation in each subscale includes the students' alliance (solidarity) (questions 1 to 8), teacher support (questions 9 to 16), the mental engagement of the students' in-class activities (questions 17 to 24), study and investigation in assignments (questions 25 to 32), goal orientations (questions 23 to 40), collaboration (questions 41 to 48), and justice and fairness (questions 49 to 54). It is designed according to the Likert scale, with a minimum score of 54 and maximum of 270. Nikdel et al. ( 24 ) reported an alpha Cronbach coefficient of 0.79 for the questionnaire. In the present study, the Cronbach's alpha coefficient was 0.87 for the questionnaire.

### Statistical analyses

Data were analyzed using descriptive and inferential statistics such as mean, standard deviation, multivariate analysis of covariance (MANCOVA), and analysis of covariance (ANCOVA). SPSS version 22.0 was further used for analyzing the data.

### Ethical considerations

The study was approved by the Ethics Committee of Islamic Azad University-Ahvaz Branch (code: IR.IAU.AHVAZ.REC.1399.006).

## Results

Table 1 presents the mean and standard deviation (SD) of the studied variables in the experimental and control groups in the pre-test and post-test. 

**Table 1:** Mean and standard deviation of the dependent variable in the experimental and control groups in pre-test and post-test

Variables	Components	Phase	Flipped classroom (Experimental)	Traditional teaching (Control)
			Mean ± SD	Mean ± SD
Perception of class	Alliance	Pre-test	29.61 ± 5.73	30.66 ± 4.66
	Support		26.16 ± 6.71	28.55 ± 4.97
	Engagement		23.44 ± 5.70	26.44 ± 7.46
	Study		27.05 ± 6.73	29.66 ± 5.36
	Orientation		31.11 ± 5.24	33.72 ± 4.22
	Collaboration		30.11 ± 4.40	32.61 ± 5.11
	Justice		23.61 ± 4.25	24.33 ± 4.22
	Alliance	Post-test	29.94 ± 4.13	29.55 ± 4.87
	Support		30.33 ± 4.37	27.77 ± 4.27
	Engagement		26.94 ± 3.88	23.88 ± 4.75
	Study		29.16 ± 4.21	27.33 ± 5.28
	Orientation		34.94 ± 3.09	30.94 ± 3.90
	Collaboration		34.72 ± 2.82	30.50 ± 4.31
	Justice		26.22 ± 3.31	21.50 ± 4.46
Self-determination	Autonomy	Pre-test	32.22 ± 6.05	33.89 ± 6.16
	Competency		30.16 ± 6.02	31.83 ± 6.16
	Relatedness		38.66 ± 6.00	42.77 ± 6.69
	Autonomy	Post-test	37.22 ± 7.38	34.88 ± 7.60
	Competency		34.22 ± 6.42	32.44 ± 5.58
	Relatedness		47.66 ± 4.33	41.38 ± 5.22

To investigate the impact of the flipped classroom on the components of class perception (alliance (solidarity), support, engagement, study, orientation, collaboration, and justice) and the components of the basic needs of self-determination (autonomy (independence), competence, and relatedness), we employed the multivariate analysis of covariance (MANCOVA). Levene's test results in investigating the homogeneity in the variance of dependent variable components in the groups (Table 2) indicated that the components' variance was equal in the groups. Moreover, the test results of the homogeneity slope of pre-test and post-test regression investigation in the groups are presented in Table 3. The results obtained from the Ljung–Box test to survey the equality of the covariances matrix in the dependent variables of the classroom perception components of the groups demonstrated that the covariance matrix of the dependent variables was equal in the two groups (P= 0.745, F= 0.282, BoX M = 0.845). Also, results obtained from the Ljung–Box test to survey the equality of the covariances matrix in the dependent variables of the self-determination components of the groups demonstrated that the covariance matrix of the dependent variables was equal in the two groups (P= 0.798, F= 0.268, BoX M = 0.834).

**Table 2:** The results of Levene's test for homogeneity of variance of dependent variable components

Variables	F	df1	df2	P
Alliance	3.30	2	51	0.078
Support	0.68	2	51	0.511
Engagement	0.95	2	51	0.392
Study	2.55	2	51	0.084
Orientation	0.08	2	51	0.923
Collaboration	1.23	2	51	0.138
Justice	1.68	2	51	0.195
Autonomy	4.20	1	34	0.062
Competency	3.58	1	34	0.061
Relatedness	1.94	1	34	0.153

**Table 3:** The results of the homogeneity of pre-test and post-test regression slope in the experimental and control groups

Source of changes	Variables	F	P
Group ^*^ Pre-test	Alliance	2.82	0.109
	Support	3.08	0.068
	Engagement	1.57	0.220
	Study	2.57	0.067
	Orientation	1.90	0.160
	Collaboration	1.41	0.252
	Justice	1.17	0.316
	Autonomy	2.99	0.064
	Competency	1.44	0.246
	Relatedness	3.10	0.062

The results of Bartlett's test for examining sphericity or meaningfulness of the relationship between the components of self-determination (basic needs) (P= 0.023, df= 2, and X^2^= 6.29) and components of class perception (P= 0.020, df= 2, and X^2^= 6.89) showed that the relationship between these variables was significant. Besides, multivariate analysis of covariance (MANCOVA) revealed a significant difference between the two groups in the components of self-determination (P= 0.0001 and Wilk's Lambda= 0.228) and class perception (P= 0.0001 and Wilk's Lambda= 0.123) (Table 4).

**Table 4:** Wilks' Lambda and Partial Eta-Square of the studied variables

Variables	Wilk's Λ	df1	df2	F	P	partial η^2^	Power
Perception of class	0.344	28	48	3.43	0.000	0.565	1.00
Self-determination	0.228	28	48	3.17	0.000	0.523	1.00

The F-ratio of analysis of covariance (ANCOVA) used for the dependent variables indicated a significant difference between the two groups in terms of class perception and self-determination (Table 5).

**Table 5:** The results of analysis of covariance (ANCOVA) for post-test scores of the variables

Variables	SS	df	MS	F	P	Partial η^2^
Perception of class	2010.704	1	2010.704	1.284	0.041	0.089
Self-determination	675.00	1	675.00	1.38	0.048	0.103

## Discussion

The present study aimed to compare the effectiveness of flipped teaching and traditional teaching on the components of self-determination and class perception of among university students. The findings demonstrated a significant difference between the mean scores of self-determinations in the two flipped classroom and traditional teaching groups. This finding is consistent with the research results of Oliveira Fassbinder et al. ( 5 ), Narendran et al. ( 9 ), and Zainuddin and Perera ( 15 ). As to these findings, it is worth noting that the students feel more confident and competent in flipped classrooms when taking part in classroom activities. Because before going to the classroom, they have already read the content of the courses and got prepared; they have done the learning activities meaningfully, thought about them, and obtained some self-learning opportunities. Furthermore, the flipped classroom creating a flexible student-centered learning environment improves the learners' independence to train them to learn independently at their own pace, so that they feel needed to investigate their knowledge as a university student independently. In this case, they will not invariably rely on their instructors, having more motivation to interact with peers and engage in the learning process ( 15 ). Three psychological needs, including independence, competence, and communication, in a learner-centered environment such as the flipped classroom, provide an incentive for the students in the realm of learning activities, determining the students' level of effort, activity, attention, and concentration ( 9 ).

According to the research findings, it can be concluded that the flipped classroom is where intra-class education changes. In the conventional classes, the class time is often used in the form of lectures, mainly focusing on conveying knowledge. Still, in the flipped classroom, face-to-face learning is combined with the online learning experiences and teaching strategies during the class period; for example, teamwork in the classroom exercises is employed to support the students' learning process. This model creates a learning environment focusing on communication between the students, the students and teachers, and between teaching and learning. As a student-centered method, it has more oversight of students' learning activities, and both student and teacher are responsible for the learning process ( 25 ).

Also, the findings indicated a significant difference between the mean scores of class perception in the two flipped classroom and traditional teaching groups. This finding is consistent with the results of Unal and Unal ( 26 ), Atkins ( 27 ), Blair et al. ( 28 ), and Vaezi et al. ( 29 ). To explain these discoveries more specifically, we can say that the flipped classroom pattern improves the learners' perception using active learning techniques. Active learning includes a set of strategies that involve the learner in the learning process, enabling him/her to deepen his/her learning. In that regard, Chen Hsieh et al. ( 30 ) believe that the flipped classroom environment provides an active and interactive learning environment where instructors guide the learners to apply the concepts and have a creative engagement with subjects. To be more specific, first, they choose the content or educational materials for the flipped classroom carefully and concisely to achieve the best learning outcomes. Videos or tasks should be concise but comprehensive to keep the students engaged and motivated. Then, instructors should ensure that students are aware of their role in the flipped class well, informing them of their responsibilities and learning objectives. Ultimately, educators should change their teaching activities in the flipped classroom to gather the learners of different levels. This method maximizes the flipped classroom approach's potentials and helps make learning and participation meaningful for all learners ( 31 ). 

In this approach, content presentation in the classroom is avoided, and instructors provide class activities by teaching the learners how to discover the cause of their issues and apply information in their real life. Flipped classroom strategies implementation increases the learners' understanding of the importance of pre-class activities and reinforcing them within the class. This pedagogy allows the teachers to spend more time teaching in the class ( 32 ). Lento ( 33 ) indicated that learners used active strategies such as debating current topics, case studies, case analysis, concept map development, and small group discussions during the class time in the flipped classroom. This education provides the instructors with the ability to engage the learners at the higher levels of Bloom's taxonomy, including creation (combination), application, and analysis. Accordingly, the lower levels of cognitive works (i.e., understanding and knowledge) are finished outside the class, and the higher levels of thinking are happening within the class with the teacher's guidance ( 34 ). Moreover, project-based learning activities in the flipped classroom help increase the learners' understanding of the content, simultaneously encouraging them to be more involved in their activities. Doing homework, repetition, and practice, question asking and answering, and discussions on educational topics are a part of class activities that replace teaching in the class. This change to the arrangement leads to class dynamism, increased motivation, and deeper learning ( 35 ). 

## Conclusion

According to the research findings, holing training courses for professors and students regarding the flipped classroom, its importance and impact on learning and education, and the means of its implementation is recommended. The flipped classroom implementation also requires computers, flash memory, high-speed Internet, and other digital devices. Before the class, we should make sure that all these devices are available. Due to the students' unfamiliarity with this teaching method, teachers should have more control over their performance in the first weeks of the flipped class.
